# Nitrate of Silver in Bed Sores

**Published:** 1843-05

**Authors:** 


*Nitrate of Silver in bed sores.*—Mr. Henry Jackson, in a paper read before the Sheffield Medical Society, extolled the efficacy of the nitrate of silver for the cure of bed sores. He mentioned one case in which "all the known remedies had been tried without avail," and in which a solution of nitrate of silver ten grains to the ounce, applied by means of a camel-hair brush, over every part exhibiting the slightest appearance of inflammation, two or three times a day, until the skin became blackened, and afterwards occasionally, answered perfectly,—*Prov. Med. Journ.*. Dec. 31, 1842.—*Amer. Journ.*

